# Sex and Response to Cardioprotective Conditioning Maneuvers

**DOI:** 10.3389/fphys.2021.667961

**Published:** 2021-05-14

**Authors:** Giulia Querio, Federica Geddo, Susanna Antoniotti, Maria Pia Gallo, Claudia Penna

**Affiliations:** ^1^Department of Life Sciences and Systems Biology, University of Turin, Turin, Italy; ^2^Department of Clinical and Biological Sciences, University of Turin, Turin, Italy

**Keywords:** cardioprotection, ischemic heart disease, estrogen, sex, conditioning, gender, reperfusion injury

## Abstract

Ischemic heart disease (IHD) is a multifactorial pathological condition strictly related to genetic, dietary, and lifestyle factors. Its morbidity and mortality rate represent one of the most important pathological issues that today involve younger people in a stronger way than in the past. IHD clinical outcomes are difficult to treat and have a high economic impact on health care. So prevention of this pathological condition through cardioprotective maneuvers represents the first line of intervention, as already underlined by several animal and human studies. Even if the time of intervention is important to prevent severe outcomes, many studies highlight that sex-dependent responses are crucial for the result of cardioprotective procedures. In this scenario sexual hormones have revealed an important role in cardioprotective approach, as women seem to be more protected toward cardiac insults when compared to male counterparts. The aim of this mini review is to show the molecular pathways involved in cardioprotective protocols and to elucidate how sexual hormones can contribute in ameliorating or worsening the physiological responses to IHD.

## Introduction

Ischemic heart disease (IHD) is a pathological condition characterized by reduced or absent blood flow in coronary arteries due to total or partial occlusion of these vessels by atherosclerotic plaque or blood clots formation. This condition causes improper supply of oxygen and nutrients to myocardial tissue, and, depending on ischemia duration, myocardial cell death can occur. Among all cardiovascular diseases (CVD), IHD is the leading single cause of death in Europe: IHD mortality represents 19% of deaths among men and 20% of deaths among women (Kuznetsova, 2018). Emerging evidences support differences in risk factors, symptoms, and outcomes of IHD between sexes in an age-related trend (Pagliaro et al., 2020). Data from the CVD statistics point out that IHD is the leading cause of death in men (16%) and women (11%) under 75, while it is the single cause of premature mortality in men (16%) under 65 and the second in women compared for the same age ranges (Wilkins et al., 2017). Ischemia can also be induced during cardiac surgery where controlled periods of cardiac arrest allow and optimize critical interventions.

Important cellular modifications during prolonged ischemia are linked to mitochondrial dysfunction and reduced ATP availability and are strictly related to intracellular Ca^2+^ impairment as already reviewed elsewhere (Garcia-Dorado et al., 2012). Furthermore, it is now well established that reperfusion, that is, the restoration of blood flow, causes further damage of cardiac tissue, maintaining intracellular Ca^2+^ overload that exacerbates the harmful effects induced by ischemia and activates calpain-mediated proteolysis, leading to the condition commonly designated as “reperfusion injury” (Garcia-Dorado et al., 2012; Inserte et al., 2012; Kalogeris et al., 2012).

According to the phenomena described, it is accepted nowadays that ischemia reperfusion injury (IRI) is characterized by detrimental effects of both ischemia and reperfusion. From a clinical perspective, even if restoration of blood flow could be dangerous, it represents the only possible intervention to have better survival chance when coronary occlusion occurs, and researches are moving toward new insights to reduce IRI and improve cardiac outcomes after prolonged ischemia.

Experimental and clinical studies have underlined sex differences in response to IRI, with women showing most detrimental outcomes, probably due to improper diagnosis and poorer prevention procedures than male counterparts (Garcia et al., 2016). IHD shows indeed different onset and clinical pictures in the two sexes, and the underestimated risk for female patients could represent the cause of poorer outcomes (Maas and Appelman, 2010). Moreover, studies are addressed to the development of therapeutic approaches in order to reduce or prevent detrimental effects of IRI in both pathological condition and cardiac surgery (Penna et al., 2015). So in the following paragraphs, we will discuss some cardioprotective maneuvers, in particular pre-conditioning (PreC), post-conditioning (PostC), and remote conditioning, that will be presented with a focus on sexual differences to these interventions, emphasizing the need for a sex-dependent approach in cardioprotection.

## Sex Differences in Physiological Cardioprotection

Nowadays it is largely accepted that there are different IRI manifestations in male and female subjects, among all ages and outcome of cardiac injury. Differences studied and results obtained suggest a possible role of sexual hormones in several animal models.

Despite the traditional dualism of estrogens and androgens in conditioning the cardioprotective responses, mainly highlighted by experiments from animal models, human studies and clinical data show a more complex scenario. In this section, cardioprotective pathways activated by both estrogens and androgens are presented, with the aim to underline the weakness of sex-adapted cardioprotective strategies only focused on these mechanisms.

Estrogen binds to different receptors: estrogen receptor-α (ER-α), estrogen receptor-β (ER-β), or G-protein coupled estrogen receptor (GPR30 or GPER). Even if ER-β has a strategical role in cardioprotection, the involvement of ER-α against IRI effects is still controversial; nevertheless, consistent data suggest that estrogen-mediated protective response may rely on both ERs (Murphy and Steenbergen, 2007b; Deschamps et al., 2010). Several pathways activated by this hormone can be seen as the opposite to those activated during ischemia or reperfusion. In fact, estrogen is involved in the regulation of ions transporters and exchangers: it induces S-nitrosylation of L-type Ca^2+^ channels with a reduction of Ca^2+^ loading, and it regulates Ca^2+^ uptake in mitochondria through the extracellular signal-regulated kinases (ERK1/2) (Iorga et al., 2017). Despite reactive oxygen species (ROS) increase being directly related to ischemia duration, it has been demonstrated that estrogen increases mitochondrial biogenesis and reduces ROS production in these cellular compartments. The hormone is also involved in the upregulation of nitric oxide synthases (NOS), through PI3-K pathway, with the consequent rise in NO production that has a primary role in activating protein kinase G (PKG) that enhances K_ATP_ channels activity and inhibits mPTP opening. Evidences show that mitochondrial preservation induced by estrogen could also be mediated by STAT3 activation through tumor necrosis factor receptor 2 (TNFR2) (Wang et al., 2008). Furthermore, estradiol decreases connexin-43 (Cx-43) dephosphorylation, which has been shown to be cardioprotective toward IRI (Murphy and Steenbergen, 2007a,b; Wang et al., 2020).

Cardioprotection in female hearts involves also endogenous antioxidant systems: catalase, superoxide dismutase (SOD), glutathione (GSH), and GSH peroxidase (GPx) are highly expressed if compared to male counterparts (Casin and Kohr, 2020). Moreover, female cardiomyocytes show high ascorbate redox homeostasis and enhanced nitrate-to-nitrite conversion that elevates NO production (Lim et al., 2009).

Other animal studies centered their work in the evaluation of the effect of testosterone in cardioprotection, and like estrogen, androgens can activate both genomic and non-genomic responses by binding to androgen receptors (AR) (Lucas-Herald et al., 2017).

There are several factors through which androgens may have a role in cardioprotection (Bell et al., 2011; Pongkan et al., 2015, 2016; Lucas-Herald et al., 2017). In fact, testosterone is involved in physiological mitochondrial ROS generation (Pagliaro and Penna, 2015); it is also able to activate the PI3-K pathway increasing endothelial NOS (eNOS) activity and NO production. Testosterone is involved in the upregulation of sarcoplasmic reticulum Ca^2+^ release channels (SERCA) and activation of L-type calcium channels, and it has been observed that in severe ischemic condition it may contribute to Ca^2+^ overload suggesting that specific context may modulate the response to this sexual hormone (Murphy and Steenbergen, 2007a; Wang et al., 2008). The positive or negative roles of androgens in cardiac tissue are under investigation, but it is already known that testosterone can be converted to estrogen by aromatase, and through this modification it can activate cardioprotective effects induced by the other sexual hormone.

So reducing the cardioprotective difference between the two sexes relying only on sexual hormones could be limiting in the management of IRI, and it is not supported by pharmacological interventions, with synthetic estrogens that demonstrated detrimental effects in postmenopausal women after IRI (Hulley et al., 1998; Anderson et al., 2004; Sivasinprasasn et al., 2016). Furthermore, new insights underline the involvement of several determinants in male and female manifestations of IRI, among which are anatomical, physiological, and genetic factors (Luczak and Leinwand, 2009; Regitz-Zagrosek and Kararigas, 2017; Stone et al., 2019; Litviňuková et al., 2020).

## Sex and Cardioprotective Maneuvers

Regarding the deleterious effects induced by IRI, future challenges for clinical procedures are addressed to the reduction of reversible or not reversible injury of cardiac tissue and the enhancement of cardioprotection. Conditioning maneuvers figure as intriguing protocols that revealed successful results in animal models of IRI, but their potential application to humans seems to underline some difficulties. In particular, PreC is not applicable in pathological settings because of ischemic unpredictability, and only a few clinical trials develop this protocol in cardiac surgery, while PostC seems to be more applicable after ischemic injury even if the window of protection occurs only a few minutes after reperfusion (Kloner and Rezkalla, 2006; Vinten-Johansen et al., 2007). Remote conditioning probably is the most plausible procedure to induce cardioprotection in clinical settings, and several clinical trials are developed to study the advantageous use of this maneuver (Candilio et al., 2011; Pedersen et al., 2018; England et al., 2019). There are plenty of data focusing on the cardioprotective effects of conditioning maneuvers on animal models, and only a few clinical trials developed in humans show contrasting results underlining the difficult translatability of these protocols to clinics (Kloner and Rezkalla, 2006; Peart and Headrick, 2009; Heusch, 2013). The following paragraphs show different sex responses to conditioning procedures in animal models, focusing on possible application to humans. Human studies and most relevant clinical trials based on the effect of conditioning are summarized in Table 1.

**TABLE 1:** Human studies and most relevant clinical trials based on the effect of conditioning maneuvers and their effect in the two sexes.

Intervention	Cardioprotective maneuver	Effect of the treatment	Sex differences	References
Coronary angioplasty for acute myocardial infarction	PostC	Reduction in infarct size and attenuation of no reflow	No sex differences studied	Staat et al., 2005
Primary percutaneous coronary intervention in STEMI	Combination of RIC and PostC	Improvement of myocardial salvage index. No differences in infarct size and microvascular occlusion	No sex differences studied	Eitel et al., 2015
Primary percutaneous coronary intervention in STEMI	RIC	Improvement of myocardial salvage index	No significant sex differences	Sloth et al., 2015
Elective coronary bypass grafting	RIC	No differences in biomarkers release	No interaction between cardioprotection and sex	Kleinbongard et al., 2016
Primary percutaneous coronary intervention in STEMI	RIPC	Reduction in enzymatic infarct size. Improvement of T2-weighted edema volume. ST-segment resolution > 50%	No sex differences studied	Crimi et al., 2013

*STEMI, ST-segment elevation myocardial infarction; PostC, post-conditioning; RIC, remote ischemic conditioning; RIPC, remote ischemic post-conditioning.*

### PreC

Among cardioprotective maneuvers PreC, is a possible therapeutic approach to limit the damaging effect of IRI. PreC consists of brief periods of ischemia and reperfusion (I/R) before the infarcting ischemia; it could be performed through mechanical or pharmacological stimulation or with exercise (Penna et al., 2020). According to its temporal application PreC could be used prior to cardiac surgery interventions, like coronary artery bypass graft surgery (CABG), but it is still difficult to perform PreC in the prevention of other unpredictable cardiac outcomes that involve IRI. Cardioprotection induced by PreC has been demonstrated to involve different molecular pathways that contribute to mitochondrial preservation through the inhibition of mPTP opening; among them are cGMP/PKG pathway that is involved in the production of NO through Akt/eNOS activation, the reperfusion injury savage kinase (RISK) pathway that activates ERK1/2 and GSK3β, and the survivor activating factor enhancement (SAFE) pathway that activates STAT3 responses (Heusch, 2013; Penna et al., 2015).

Given the effect of PreC, and the implications of sex hormones in cardioprotection, it could be interesting to evaluate if there are different responses to PreC in male and female subjects.

In order to assess any difference between sexes in PreC followed by ischemia, it has been evidenced in animal models that not only sex but also age influences the resulting cardioprotection: in fact, young female animals show no response to PreC presumably due to incomplete sexual maturation, while male subjects show cardioprotection after PreC at any age (Turcato et al., 2006).

Furthermore, several studies underline positive effects of PreC before IRI in males but no effects or worst injury in female subjects. Some results suggest that the female heart is physiologically protected by estrogen, and thus the effect of PreC can be insufficient to overcome that of the sexual hormone, while male subjects benefit at all ages from the treatment with PreC. To support this hypothesis, some groups underline the role of different cardioprotective mediators in PreC and their physiological expression in the two sexes: K_ATP_ channels and heat shock proteins (HSP) that are constitutively more activated in females (Ranki et al., 2002; Deschamps et al., 2010), the enzymatic or non-enzymatic capability to control increasing ROS that is more evident in female hearts (Casin and Kohr, 2020), and PI3-K/NOS pathway activation through Notch1 and GPR30 that is more expressed in female hearts (Rocca et al., 2018). On the other hand, a study by Lieder and collaborators underlines no sex differences after PreC treatment of rat hearts showing no sex-dependent differences in infarct size; the authors discuss their results underlining that diverging data in this field could be caused by animal models or different experimental protocols (Lieder et al., 2019). In this scenario, it is highly supported that female cardioprotection induced by PreC can be reached only if cardiac insults overcome a stress threshold that makes the existing estrogen protection insufficient (Song et al., 2003; Pitcher et al., 2005); lack of clinical trials or human studies that show different responses in the two sexes compels this consideration only to a theoretical field.

### PostC

Another cardioprotective intervention that can be used to reduce IRI is PostC: brief intermittent cycles of I/R mechanically or pharmacologically induced in the onset of reperfusion. PostC activates different intracellular responses; the most studied are the cGMP/PKG pathway, the RISK pathway, and the SAFE pathway that all contribute to mitochondrial preservation and limit cardiac damage (Heusch, 2013). Emerging clinical studies are addressed to translate animal model results to humans, also because PostC has a great clinical potential and its application could be promising in cardioprotective strategies (Vinten-Johansen et al., 2007). As seen in PreC, it could be reasonable to think that differences between sexes can also be found in PostC responses (Skyschally et al., 2009).

It has been observed that after PostC only males show improved post-ischemic recovery of function, and protective effects of PostC in male subjects involve different factors, such as reduced superoxide production, increased MnSOD expression, reduced Bax/Bcl-2 ratio, and reduced caspase-3 activation (Ciocci Pardo et al., 2018). Furthermore, after PostC male hearts also show higher expression in p-Akt, p-GSK3β and p-PKCε, while female expression of these intracellular mediators has no changes after the treatment (Ciocci Pardo et al., 2018). Moreover, Inserte and collaborators show that in male Sprague–Dawley rat hearts, PostC activates the cGMP/PKG pathway that is involved in cardioprotection through the delaying normalization of intracellular pH (Inserte et al., 2011). Studies on animal models show that PostC maneuvers have effects only in male hearts; lack of data in female animal models make it difficult to define clear differences between sexes.

Regarding all the molecular responses to PostC in males hearts, it could be speculated that PostC response is also strictly related to sex hormones showing that probably it activates pathways that are already highly expressed in females due to estrogen stimulation; so to have PostC cardioprotective effect, it could be reasonable that female hearts have to be exposed to higher injury (Crisostomo et al., 2006; Penna et al., 2009). Some human clinical trials and meta-data analysis underline no beneficial effect of PostC application, but the different sex response was not considered by these studies (Eitel et al., 2015; Xing et al., 2019), while other works show a positive effect of PostC in reducing infarct size, but no sex differences were outlined (Staat et al., 2005). Furthermore, some evidences also underline that the hearts of women treated with PostC showed worse outcomes compared to untreated women, pointing out the need for deeper investigations on the possible application of PostC in female patients (Shin et al., 2019).

### Remote Conditioning

Remote ischemic conditioning (RIC) is considered among cardioprotective maneuvers; it consists of brief cycles of ischemia and reperfusion in a peripheral organ or tissue (even in arms or legs), remote from the heart (Przyklenk et al., 1993). It can be induced before (remote PreC), during (remote per-conditioning), or after (remote PostC) an index ischemia (Penna et al., 2015). It is a non-invasive and low-cost procedure that can be performed through an inflating/deflating pneumatic cuff to induce 5 min cycles of ischemia/reperfusion favoring the protection induced by RIC (Hausenloy et al., 2020). Clinical benefits of this procedure are still debated (Hausenloy et al., 2019, 2020), but recent findings point out a possible role of humoral factors released after RIC that have an age- and sex-dependent protective role (Heinen et al., 2018). In particular, Heinen et al. (2018) point out a significant protective role of humoral factors derived from young males exposed to RIC in reducing infarct size, and this is probably due to the phosphorylation of GSK3β that is involved in the inhibition of mPTP through the RISK pathway. Furthermore, a recent work by Lieder et al., reported no differences in cardioprotection in a specific RIC model (Lieder et al., 2019). Clinical data suggest controversial results on the efficacy of RIC, some of them showing no cardioprotection (García Del Blanco et al., 2021), while others underlining cardioprotective effects of RIC with no sex differences (Crimi et al., 2013; Eitel et al., 2015; Sloth et al., 2015; Kleinbongard et al., 2016). Insufficient data in animal and clinical studies point out differences between sexes in cardioprotective effects of RIC, and some authors suggest estrogens as possible confounding factors that make difficult the interpretation of limited data regarding the role of RIC in female subjects (Brevoord et al., 2012; Lieder et al., 2019; Shaban and Leira, 2019).

## Conclusion

**FIGURE 1:**
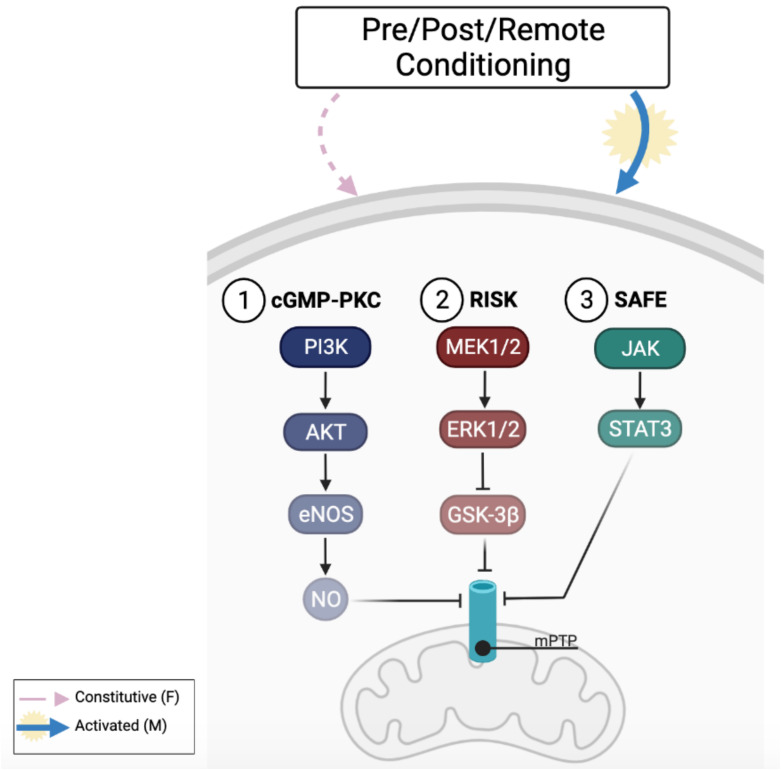
Cardioprotective pathways activated by conditioning maneuvers and sex-related response. Animal models suggest that males (M) seem to respond better to conditioning cardioprotective maneuvers through the activation of cGMP-PKC (1), RISK (2) and SAFE (3) pathways than female (F) that constitutively express these pathways. Figure created in BioRender.com.

In this brief report, we have outlined different responses in female and male hearts to IRI, and in particular we focused on a primary role of estrogens in cardioprotection. Some of the most studied cardioprotective maneuvers, in order to reduce IRI, have been described with a particular focus on females and males’ different responses. The overview outlined here shows that differences between sexes in cardioprotective interventions could be linked, but not exclusively, to the physiological role of sexual hormones that change throughout the lifespan highlighting a complex relationship toward age and sex in response to cardioprotective maneuvers. In conclusion, sex and age differences have to be considered in cardioprotection in order to optimize the clinical application of these procedures. As Figure 1 shows, we have focused on intrinsic cardioprotective mechanisms that can be elicited by conditioning maneuvers as in our opinion it is important to understand the sex-related differences in these mechanisms before moving on to testing pharmaceutical approaches. Furthermore, a deeper knowledge of the protective pathways activated by the different conditioning maneuvers in the two sexes represents a crucial point for clinical interventions.

## Author Contributions

GQ, MG, SA, and CP conceived the study and its design, and revised the manuscript for important intellectual content. GQ, MG, and CP wrote the manuscript. FG created the figure and revised the manuscript. All authors read, edited, and approved the final manuscript.

## Conflict of Interest

The authors declare that the research was conducted in the absence of any commercial or financial relationships that could be construed as a potential conflict of interest.
